# Establishment of a stellate ganglion regulation model in mice using infrared polarized light irradiation

**DOI:** 10.3389/fphys.2025.1609482

**Published:** 2025-08-21

**Authors:** Kaixuan Zhao, Haoyue Zhang, Yanbo Liu, Ying Zhou, Juan Zhi, Qianyu Wang, Dong Yang

**Affiliations:** Department of Anesthesiology, Plastic Surgery Hospital, Chinese Academy of Medical Sciences and Peking Union Medical College, Beijing, China

**Keywords:** stellate ganglion, infrared polarized light, Horner’s syndrome, heart rate, animal model

## Abstract

**Objective:**

To explore the feasibility of establishing a mouse stellate ganglion (SG) regulation model through infrared polarized light (IPL) irradiation of the SG, and preliminarily evaluate its effects on SG function and related physiological indicators. Surgery, and IPL groups, with 8 mice in each group. A ZZIR-ID therapeutic device was used to directly irradiate bilateral SG regions of IPL group mice, with wavelength 980 nm, power density 1000 mW/cm2, 10 min per session (5 min per side), every other day for 6 times. The control group received no treatment, while the.

**Results:**

Compared with the control and Sham surgery groups, the incidence of Horner’s syndrome in the IPL group increased significantly (P < 0.05), manifesting as bilateral ptosis and enophthalmos, lasting about 2 h. Immediately after treatment, eye temperature in the IPL group increased significantly compared to pre-treatment (P < 0.05). Heart rate in the IPL group decreased significantly 30 min post-treatment compared to pre-treatment (P < 0.01), lasting 1–2 h. There was no statistically significant difference in weight changes between groups (P > 0.05). In all treated mice, the characteristic signs of Horner’s syndrome developed within minutes of starting IPL exposure, reached their peak intensity between 1.5 and 2.5 h, and resolved completely within 3–4 h of the 10-min irradiation session.

**Conclusion:**

IPL irradiation of SG can effectively induce Horner’s syndrome in mice, elevate eye temperature, and reduce heart rate. These findings suggest IPL as a potential method for modulating SG activity in preclinical models.

## 1 Introduction

The stellate ganglion (SG) is the cervical segment of the sympathetic nervous system, located in the lower neck, formed by the fusion of the 7th cervical and 1st thoracic sympathetic ganglia. Anatomical studies have shown that the SG sends sympathetic nerve fibers to innervate ipsilateral upper limbs, heart, lungs, thyroid and other organs, playing an important role in regulating vasomotor, glandular secretion and organ function in these regions (Witt et al., 2017). In recent years, with the deepening understanding of SG function, its role in the pathogenesis of various diseases has received increasing attention. A large body of research indicates that SG dysfunction can lead to sympathetic-vagal imbalance, promote pain and inflammatory responses, aggravate tissue damage, and participate in the occurrence and development of intractable pain, stress disorders, cardiovascular events and other diseases (Lipov et al., 2020; Lipov et al., 2022; Wen et al., 2021). Therefore, SG has become a potential therapeutic target for these diseases, and new strategies to alleviate related symptoms and signs and improve prognosis by regulating SG activity are highly anticipated.

Animal models of SG blockade or resection are important means to explore SG function and its regulatory effects. Traditional methods mainly use local anesthetic injection or surgical resection of SG to establish animal models. However, local anesthetic SG blockade may cause complications such as pneumothorax and local anesthetic toxicity, and the blockade effect is affected by factors such as drug type, dose, and injection site, resulting in low standardization (Piraccini et al., 2023). Although surgical resection of SG provides thorough blockade, it is associated with greater trauma and more complications, and its irreversibility limits its application in mechanistic studies. Moreover, large and medium-sized animal models are costly to construct, while the anatomical position of SG in rodents like mice is relatively deep, making surgery challenging and inconvenient for research work. Therefore, there is an urgent need for a new type of SG functional regulation model that is simple to operate, effective, minimally invasive, and repeatable.

In recent years, low-level light therapy (LLLT) has been widely applied and studied in multiple biomedical fields due to its non-invasive, safe, and repeatable characteristics. Studies have shown that percutaneous low-level light therapy (LLLT) near the SG region or linearly polarized near-infrared irradiation can effectively relieve pain in patients with neuropathic pain and affect heart rate variability, possibly related to light irradiation regulating SG activity (Hashimoto et al., 1997; Liao et al., 2017). However, research on directly using light irradiation to establish SG regulation model in mice has not been reported.

IPL is a special near-infrared light with stronger penetration, polarization degree and photon energy utilization compared to conventional light sources, showing good effects in tissue repair, pain rehabilitation and other aspects (Hamblin, 2017). Our team’s previous study established a stellate ganglion block model in mice using traditional methods (e.g., local anesthetic injection), confirming the feasibility of SG regulation and its effects on physiological indicators (Duan et al., 2024). Given the relatively superficial anatomical location of SG in mice, direct percutaneous IPL irradiation of SG is technically feasible.

This study intends to use a ZZIR-ID infrared polarized light therapeutic device to directly irradiate bilateral SG regions in BALB/c mice, and preliminarily explore the feasibility of IPL inducing SG functional changes and establishing a mouse SG regulation model by observing Horner’s syndrome manifestations, eye temperature, heart rate and inflammatory factor levels. The study also aims to evaluate its effects on autonomic nervous and immune functions, in order to provide new ideas and experimental evidence for the research application of IPL in the prevention and treatment of SG-related diseases.

## 2 Materials and methods

### 2.1 Experimental animals

Considering the preliminary nature of this study and the 3R principles (Replacement, Reduction, and Refinement), we used the resource equation method to estimate sample size, aiming to minimize animal use while meeting statistical analysis requirements. This method is suitable for studies with quantitative variables and is commonly used in analysis of variance (ANOVA) designs. The sample size estimation formula is E = N - K, where E is the degrees of freedom for error, N is the total sample size, and K is the number of treatment groups (K = 3 in this study). It is generally recommended that 10 ≤ E ≤ 20 to ensure sufficient statistical degrees of freedom. We chose N = 24 (E = 24–3 = 21), with 8 mice per group, slightly above the upper recommended limit, to reduce random error, enhance statistical power, and provide a buffer for potential animal losses, ensuring result reliability and stability.

Twenty-four 6–8 weeks old clean grade male BALB/c mice weighing 20–25 g were provided by the Laboratory Animal Center of Chinese Academy of Medical Sciences [Certificate No.: SCXK (Beijing) 2014-0004]. The mice were randomly divided into control group, sham surgery group and IPL group, with 8 mice in each group. Animals were housed in the clean animal facility of the Institute of Plastic Surgery, Chinese Academy of Medical Sciences, with free access to food and water, temperature (22 ± 2) °C, relative humidity (55 ± 10)%, and 12 h light/dark cycle.

This study followed the guidelines for animal care and experimentation set by the Research Animal Care Committee of the Institute of Plastic Surgery, Peking Union Medical College Hospital, Chinese Academy of Medical Sciences (Ethical approval No. 20240030112). The Institutional Animal Care and Use Committee of the Institute of Plastic Surgery, Peking Union Medical College Hospital approved these experiments. The study also adhered to the ARRIVE guidelines for *in vivo* experiments in animal research, with the experimenter’s certificate number being 1121032400006. At the end of the experiment, mice were euthanized by CO2 inhalation in accordance with institutional guidelines for animal welfare.

### 2.2 Instruments and reagents

Infrared polarized light therapeutic device: ZZIR-ID (Zhuangzhi Company, China), wavelength 980 nm, power density 1,000 mW/cm^2^. Small animal physiological recorder: RM6240E (Chengdu Instrument Factory, China). Infrared thermal imager: HIKVISION TB4117-3S (Hikvision, China), thermal sensitivity <0.05 °C. Small animal ventilator: MiniVent Type 845 (Harvard Apparatus, United States).

### 2.3 Bilateral SG region IPL irradiation method

Mice were manually restrained and irradiated using a ZZIR-ID infrared polarized light therapeutic device (Beijing Zhuangzhi Technology). The emission probe was placed vertically on the right side of the neck skin, positioned at the carotid artery pulsation point (midpoint between the lower edge of the sternocleidomastoid muscle and the skull).

IPL irradiation parameters were as follows: wavelength 980 nm, power density 1,000 mW/cm^2^, spot diameter 5 mm,10 min per session (5 min per side), every other day for a total of 6 sessions; these parameters were determined based on the manufacturer’s recommendations and preliminary experiments.

**FIGURE 1:**
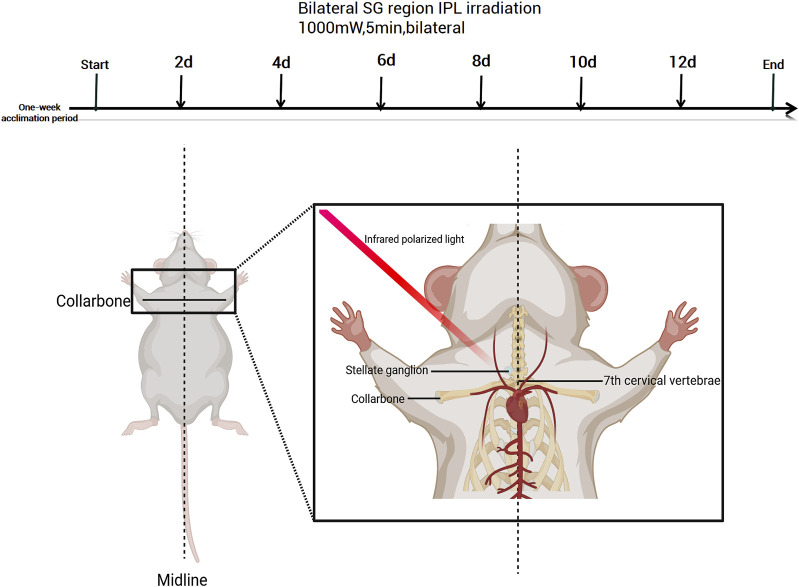
Experimental timeline and anatomical illustration of stellate ganglion irradiation in mice. Created with BioRender.com (Version available at: https://www.biorender.com/).

The control group received no treatment, while the Sham surgery group received IPL irradiation with the same parameters only on the top of the head (away from bilateral SG regions), both avoiding direct lens stimulation to the eyes (refer to Figure 1).

### 2.4 Observation indicators and methods

#### 2.4.1 Horner’s syndrome assessment

Bilateral eyes of mice were observed before and immediately after treatment, recording the incidence rate and duration of Horner’s sign (ptosis, enophthalmos). Referring to literature methods (Lin et al., 2023), ptosis severity was graded as: no ptosis (−), mild (+), moderate (++), and severe (+++). Changes of + or above were considered positive for Horner’s sign.

#### 2.4.2 Eye temperature measurement

Infrared thermal imaging of mouse eyes was performed before and immediately after treatment, analyzing the highest temperature around the orbit. Three mice were tested in each group, with each eye measured 3 times and the average value taken.

#### 2.4.3 Hart rate monitoring

Electrocardiogram of awake mice was continuously recorded for 30 min before and 30 min after each treatment using a physiological recorder, analyzing average heart rate every 5 min.

#### 2.4.4 General condition observation

General state, activity level and weight changes of mice were recorded daily.

### 2.5 Statistical analysis

SPSS 20.0 software (SPSS Inc., Armonk, New York, NY, United States) was used for statistical analysis. Before statistical analysis, all measurement data (e.g., eye temperature and heart rate) were assessed for normality using the Shapiro-Wilk test. If the data followed a normal distribution (P > 0.05), parametric tests were used; otherwise, non-parametric tests were applied. Upon testing, all data in this study were normally distributed. Measurement data were expressed as means ± standard deviation (x ± s), and between-group comparisons were conducted using one-way analysis of variance (ANOVA). If differences were significant (P < 0.05), pairwise comparisons were further performed using the LSD-t test. Within-group comparisons (e.g., changes in eye temperature and heart rate before and after irradiation) were analyzed using paired t-tests. Count data are expressed as number of cases (composition ratio), and comparisons between groups were performed using χ2 test or Fisher’s exact probability method. *P* < 0.05 was considered statistically significant.

## 3 Results

### 3.1 IPL irradiation induces Horner’s syndrome

**FIGURE 2:**
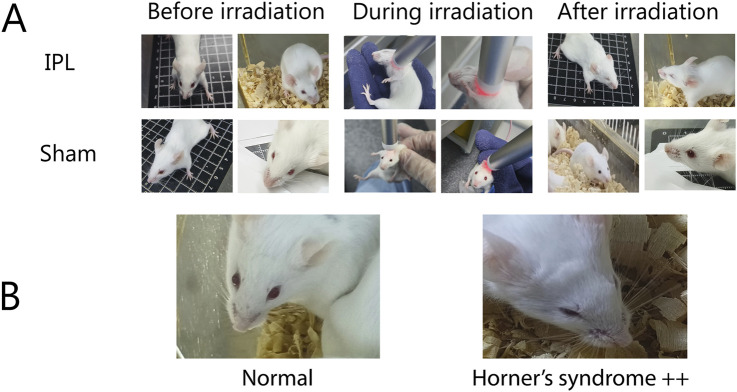
**(A)** Comparison of IPL and Sham treatments on mice, **(B)** demonstration of Horner’s syndrome.

Before treatment, no Horner’s syndrome manifestations were observed in any group. Mice in the IPL group exhibited bilateral Horner’s sign immediately after the first irradiation, manifesting as ptosis and reduced corneal exposure (Figure 2). Crucially, this effect was consistently and fully reversible. The signs typically persisted for approximately 2–3 h post-irradiation before gradually resolving, with all animals returning to a normal state well before the next treatment session at 48 h. As the number of irradiations increased, the incidence rate of Horner’s sign in the IPL group remained at a consistent 100%, with no evidence of tachyphylaxis or altered severity. After the last treatment, the 100% (8/8) incidence rate in the IPL group was significantly higher than 0% (0/8) in the control group and 12.5% (1/8) in the Sham surgery group.

### 3.2 IPL irradiation elevates eye temperature

Before irradiation, there was no statistically significant difference in eye temperature between the IPL and Sham groups (35.913 °C ± 0.203 °C vs. 35.925 °C ± 0.149 °C, P > 0.05). After irradiation, the eye temperature of mice in the IPL group increased to 36.638 °C ± 0.220 °C, which was significantly higher than before irradiation (P < 0.0001). In the Sham group, eye temperature after irradiation (36.013 °C ± 0.173 °C) showed a slight increase, but this change was not statistically significant compared to before irradiation (P > 0.05).

The increase in eye temperature was more pronounced in the IPL group (0.725 °C) compared to the Sham group (0.088 °C). This difference in temperature change between the two groups was statistically significant (P < 0.0001), indicating that IPL irradiation had a greater effect on elevating eye temperature than the Sham procedure.

**FIGURE 3:**
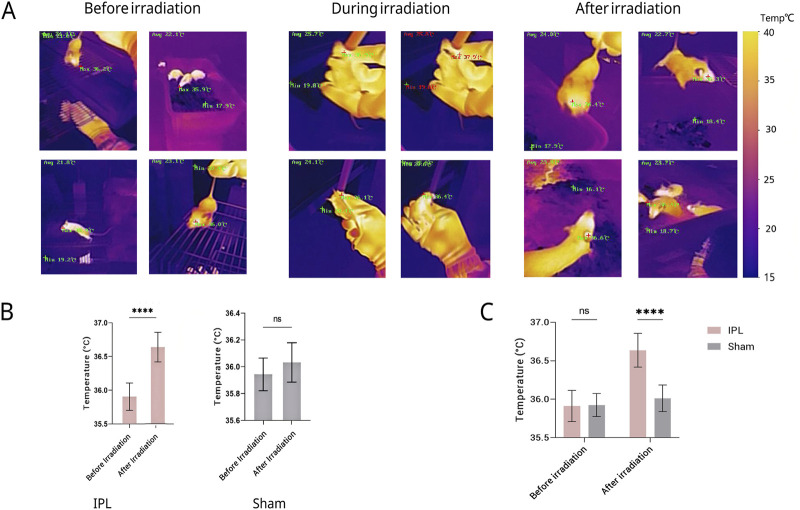
Changes in eye temperature of mice across different groups and time points. **(A)** Representative infrared thermal images of mouse eyes before, during, and after irradiation. A representative color temperature scale bar is provided on the right, mapping the pseudo-color display to temperatures in degrees Celsius. Quantitative maximum (Max) and minimum (Min) temperatures are also displayed on each image. **(B)** Quantitative comparison of eye temperature before and after irradiation. **(C)** Results show that eye temperature in the IPL group increased significantly compared to pre-treatment immediately after the first irradiation (P < 0.05). After the final treatment, eye temperature in the IPL group was significantly higher than both the control and Sham surgery groups (P < 0.05). No significant changes were observed in the control and Sham surgery groups throughout the experiment. Data are presented as mean ± SD. ****P < 0.0001.

These results suggest that IPL irradiation of the stellate ganglion region can effectively increase eye temperature in mice, while the Sham procedure does not produce a significant change. This provides evidence for the physiological effects of IPL on the autonomic nervous system, particularly its influence on ocular thermoregulation. See Figure 3.

Furthermore, the possibility that the observed ocular temperature elevation resulted from direct thermal effects of the infrared light source was carefully examined.In our experiment, the maximal ocular temperature increase in the IPL group (∼0.7 °C) was modest and far below the temperature of the irradiated neck region.The Sham group, exposed to the same power density at the cranial vertex, displayed only a minimal (∼0.1 °C) ocular temperature rise and no sustained physiological changes.These observations strongly argue against a direct heating artefact and support the interpretation that the change in ocular thermoregulation reflects modulation of sympathetic outflow rather than passive warming.

### 3.3 IPL irradiation reduces heart rate

**FIGURE 4:**
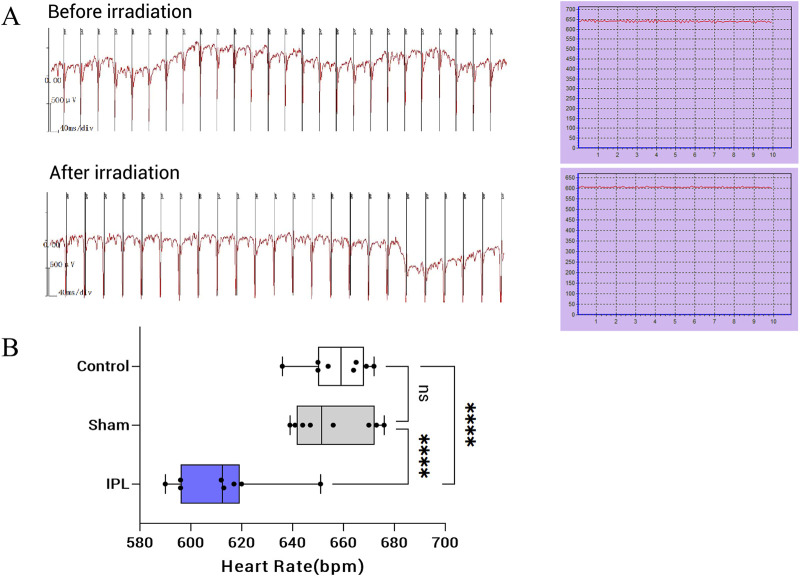
Effects of infrared polarized light (IPL) irradiation on heart rate in mice. **(A)**: Representative electrocardiogram (ECG) recordings and heart rate curves before (top) and after (bottom) IPL irradiation. The ECG shows a decrease in heart rate following IPL treatment. **(B)**: Box plot comparing heart rates among Control, Sham, and IPL treatment groups. The IPL group shows a significant reduction in heart rate compared to both Control and Sham groups. Each box represents the interquartile range, with the median indicated by the horizontal line. Whiskers extend to the minimum and maximum values, excluding outliers. Individual data points are overlaid on each box plot. ****P < 0.0001.

Before treatment, heart rate levels were similar between the three groups, with no statistically significant difference (P > 0.05). At 30 min after the last irradiation, heart rate in the IPL group decreased to (611.9 ± 22.4) beats/min, significantly slower than both the Sham (655.8 ± 22.4) beats/min and control (657.5 ± 22.4) beats/min groups (P < 0.001 for both comparisons). The difference between the IPL group and the Sham group was 43.88 beats/min (q = 7.842, DF = 21), while the difference between the IPL group and the control group was 45.63 beats/min (q = 8.155, DF = 21). There was no significant difference in heart rate between the Sham and control groups (difference of 1.75 beats/min, q = 0.3128, DF = 21, P > 0.05). These results indicate that IPL treatment significantly reduced heart rate compared to both Sham treatment and no treatment, while Sham treatment had no significant effect on heart rate compared to the control group. See Figure 4.

### 3.4 Comparison of general conditions between groups

**FIGURE 5:**
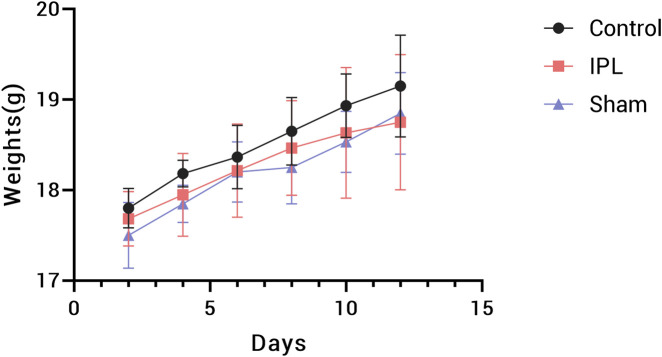
Body weight changes in mice during the 14-day experimental period. Data points represent mean weights for Control (black circles), IPL treatment (red squares), and Sham (blue triangles) groups. Error bars indicate standard deviation. n = 8 mice per group.

During the experiment, mice in all groups maintained good general condition and spontaneous activity, with gradual weight gain and no statistically significant overall difference (P > 0.05). Notably, mice in the IPL group showed reduced activity a few seconds after irradiation began and were able to maintain a fixed posture, being more cooperative than the Sham group, possibly due to the sedative effect of stellate ganglion irradiation. Figure 5.

## 4 Discussion

This study pioneered a novel stellate ganglion (SG) functional regulation model in BALB/c mice using bilateral infrared polarized light (IPL) irradiation directly on the SG. Compared to control and sham surgery groups, IPL irradiation significantly increased the incidence and severity of Horner’s syndrome in mice, accompanied by autonomic nervous system responses such as elevated eye temperature and decreased heart rate, indicating effective bilateral SG function inhibition. These physiological effects largely parallel those observed in clinical SG block (SGB) using local anesthetics or other methods (Duan et al., 2024; Wulf and Maier, 1992; Ghai et al., 2016; Feigl et al., 2007). Importantly, these physiological effects were transient and fully reversible: Horner’s signs, ocular temperature elevations, and heart rate reductions dissipated within a few hours after each treatment, with animals returning to baseline well before the next session.

Horner’s syndrome, a classic indicator of SGB efficacy, arises primarily from dysfunction of postganglionic sympathetic fibers on the blocked side, leading to a constellation of clinical manifestations including ipsilateral ptosis, enophthalmos, and miosis (Reede et al., 2008). Numerous studies have demonstrated that both percutaneous low-intensity laser irradiation of the neck and local anesthetic SGB can elicit pronounced Horner’s syndrome along with autonomic responses like regional temperature elevation and heart rate reduction (Amhaz et al., 2013; Chen et al., 2015; Kirkpatrick et al., 2023). The striking resemblance between the IPL-induced SG regulation model in mice and human study results, in terms of Horner’s sign presentation, eye temperature, and heart rate changes, underscores the model’s clinical relevance in simulating human SG functional status and its regulatory effects.

As a form of near-infrared light, IPL’s potent tissue penetration capabilities endow it with diverse photobiomodulatory effects in biological tissues. When irradiating the SG, IPL may potentially affect adjacent structures such as the vagus nerve or carotid artery. However, the wavelength (980 nm) and power density (1,000 mW/cm^2^) used in this study offer strong tissue penetration, possibly limiting direct effects on neighboring structures. Future studies could further exclude non-specific effects through anatomical and functional assessments.

The physiological effects observed in this study invite an inquiry into the underlying molecular mechanisms. The principles of photobiomodulation suggest that near-infrared light is primarily absorbed by mitochondrial chromophores, most notably cytochrome c oxidase, leading to an increase in cellular ATP production and modulation of intracellular signaling pathways (Hamblin, 2017; Chung et al., 2012). Such alterations in the metabolic state of neurons can profoundly influence their electrophysiological properties and signaling functions. To further investigate this within our model, supplementary biochemical analyses were performed on stellate ganglion tissue following the IPL intervention. These analyses revealed a discernible increase in ATP concentration within the ganglion tissue of the treated group compared to the sham control. Concurrently, we observed a reduction in the stored levels of the sympathetic neurotransmitter, norepinephrine, within the ganglia, suggesting an alteration in its release or turnover dynamics. These preliminary findings are consistent with the hypothesis that SG-IPL may modulate sympathetic outflow by directly affecting both the energetic and neurochemical status of the ganglion neurons. This aligns with broader research indicating that autonomic neuromodulation can have significant downstream consequences on the function of innervated organs (Pavlov and Tracey, 2017).

Our finding of increased ATP production is consistent with the established primary mechanism of PBM, wherein the activation of mitochondrial cytochrome c oxidase stimulates ATP synthesis. However, this metabolic enhancement is rarely an isolated event. It is often accompanied by a transient increase in reactive oxygen species (ROS) and alterations in calcium ion influx. These initial events can trigger a cascade of downstream signaling pathways that extend beyond simple energy metabolism, influencing broader cellular processe (Pchelin et al., 2023; Amaroli et al., 2019). While our study provides direct evidence for the modulation of ATP and norepinephrine, the full spectrum of IPL’s influence on the SG likely involves a more complex interplay of these factors. For instance, the observed neurochemical changes could be linked to the modulation of local inflammatory mediators or other signaling molecules within the ganglion microenvironment (Chen et al., 2011; Tsai and Hamblin, 2017; Nakase et al., 2004). Elucidating the precise hierarchy and interplay between these energetic, neurochemical, and immunological pathways represents a promising avenue for future investigation.

The resource equation method was employed to estimate sample size in this study, offering greater flexibility than traditional power analysis without requiring preset effect sizes while balancing sample size with experimental quality and animal welfare considerations. However, the limited sample size of the current study constrains the generalizability and statistical power of the results. This study did not perform a power analysis to determine effect size; future research could use larger sample sizes and Cohen’s d analysis to further validate the robustness of the results (Festing and Altman, 2002).

Given the well-documented stress sensitivity of BALB/c mice (Hull et al., 2024), we implemented rigorous measures to minimize confounding stress effects, including standardized housing conditions, experienced handlers, and minimal animal handling. The single transient ocular response observed in the sham group suggests that cranial IPL irradiation may produce minor procedural stress. However, several lines of evidence support our sham design’s validity for isolating SG-specific effects:

First, the experimental group received targeted SG irradiation without cranial exposure, while sham mice underwent cranial irradiation specifically selected to match ocular light scatter and restraint conditions without SG involvement. This design ensured comparable non-specific factors between groups while selectively activating the SG in experimental animals.

Second, while transcranial near-infrared light can modulate cortical activity (Lin et al., 2024; Salehpour et al., 2018), such effects are mechanistically distinct from the sympathetic blockade required to induce Horner’s syndrome. The complete absence of sustained Horner’s signs in sham mice (*versus* 100% incidence in SG-irradiated animals) strongly suggests that cranial irradiation does not meaningfully influence SG function.

Third, alternative control approaches (e.g., abdominal irradiation) would fail to match the critical variables of ocular exposure and restraint stress present during SG irradiation. The cranial sham procedure thus represents the most physiologically relevant control for this experimental paradigm. We acknowledge that additional controls using inactive light sources could further strengthen these conclusions in future studies. However, the marked differential outcomes between groups, combined with the anatomical and mechanistic specificity of Horner’s syndrome, provide compelling evidence that our observed effects reflect true SG modulation rather than non-specific light effects.

A consideration central to the interpretation of our findings is the design of the sham control. In this study, we selected cranial irradiation as the control procedure. Our rationale for this choice was predicated on the need to account for all potential non-specific variables inherent to the experimental paradigm. Beyond the evident factor of restraint stress, which was matched between groups, a more subtle confounder warranted careful consideration: the potential for scattered light to affect ocular structures. Given the anatomical proximity of the stellate ganglion to the head, it was conceivable that scattered photons from the active probe could elicit non-specific physiological responses in the eye. A sham procedure utilizing an inactive probe would not address this particular variable. The cranial irradiation protocol was therefore implemented to control for both restraint stress and this potential ocular light exposure, while avoiding direct irradiation of the stellate ganglion. The marked disparity in outcomes—the complete absence of sustained Horner’s syndrome or significant autonomic shifts in the sham group *versus* the consistent and profound effects in the IPL group—lends substantial support to the conclusion that our observations are attributable to the specific modulation of the SG. It is pertinent to note that in other studies from our research group applying this model, a sham control utilizing an inactive probe yielded results consistent with those of the cranial sham group presented herein, further reinforcing the specificity of the SG-targeted intervention.

The equipment used in this study has inherent limitations in thermal imaging resolution and annotation clarity, potentially affecting thermographic pattern discernment (Hu et al., 2018). While these instruments offer significant cost-benefit advantages and broad accessibility for replication in resource-limited settings, future research could adopt hyperspectral imaging systems or lock-in thermography to enhance thermal contrast resolution (Kotwal et al., 2025). Implementing machine learning-assisted thermal signal reconstruction algorithms could further address interpretation challenges due to low thermal sensitivity while retaining the practical advantages of traditional thermal imaging platforms (Liu et al., 2012).

SG dysfunction has been implicated in the pathogenesis and progression of various conditions, including complex regional pain syndrome, post-traumatic stress disorder, and arrhythmias (Shim et al., 2019; Drummond and Finch, 2004; Cathomas et al., 2019). In clinical practice, SGB serves as an effective adjuvant therapy for these disorders. However, due to species and anatomical differences, traditional human SGB techniques cannot be fully replicated in rodents. The IPL-induced SG functional regulation model not only circumvents the limitations of conventional animal models in terms of operational complexity and invasiveness but also provides a powerful tool for in-depth exploration of the pathogenic mechanisms underlying SG dysfunction-related diseases and evaluation of novel interventions. Future work can leverage this model to investigate the relationship between SG functional abnormalities and disease, further elucidate the molecular and neural mechanisms of IPL’s actions, and assess its potential applications in disease prevention and treatment.

## 5 Conclusion

Our findings demonstrate that bilateral infrared polarized light (IPL) irradiation targeted at the stellate ganglion region induces characteristic physiological responses in mice, including Horner’s syndrome manifestations, elevated ocular temperature, and reduced heart rate. These observations suggest that IPL may serve as a potential approach for modulating stellate ganglion function in murine models. However, the underlying mechanisms of these effects and their duration require systematic investigation through additional studies. Further research is also needed to evaluate the safety profile and potential clinical applicability of this technique.

## Data Availability

The datasets presented in this study can be found in online repositories. The names of the repository/repositories and accession number(s) can be found below: https://doi.org/10.5281/zenodo.13890741.
